# Complete genome sequences of nine isolates from microplastics in the Bow River, Calgary, Canada

**DOI:** 10.1128/mra.01085-24

**Published:** 2025-04-04

**Authors:** Kira L. Goff, Sneha Suresh, Jonas M. Stadfeld, Srijak Bhatnagar

**Affiliations:** 1 Enviromics Research Group, Centre for Science, Athabasca University, Athabasca, Alberta, Canada; 2 Enviromics Research Group, Department of Biological Sciences, University of Calgary, Calgary, Alberta, Canada; California State University San Marcos, San Marcos, California, USA

**Keywords:** microplastics, freshwater ecosystem, plastisphere, plastic degradation, Nanopore sequencing, surface water, sediment, River microplastics

## Abstract

We present the complete genome sequences of nine bacterial strains isolated from microplastics from water or sediments of the Bow River in Calgary, Alberta. These isolates provide insight into the freshwater microplastic microbiome and their plastic biodegradation potential.

## ANNOUNCEMENT

Microplastics are pervasive contaminants in environments throughout the globe, serving as hosts for microbial communities distinct from those in the surrounding environment (1). Here, we present genomes of bacteria isolated from microplastics in the Bow River, after the confluence with Nose Creek and Elbow River, within Calgary, Canada’s fourth largest city.

Microplastics were isolated from water (40 L) and sediment samples (0.5 Kg) (51.04344 °N, 114.01502 °W) through 20 µm filtration or CaCl_2_ density separation (1.4 Kg/L), respectively, followed by Nile red staining (2). Microplastics were rinsed twice with 1× PBS buffer and inoculated in either liquid LB (3) or R2A media (4) at room temperature (22°C). Upon visible growth (2–3 days), a 10^−4^ dilution of the liquid culture was spread-plated on the respective solid media (LB or R2A with 1.5% agar at room temperature), followed by 4–5 rounds of streaking to achieve isolation. One colony from each isolate was grown in respective liquid media for 48 hours and used for high-molecular-weight DNA extraction using the Lucigen MasterPure Complete DNA & RNA Purification Kit (Mandel Scientific) using the manufacturer-supplied protocol for Gram-positive bacteria. DNA (no shearing or size selection) was sequenced on an R10.4.1 Nanopore MinION flowcell using Native Barcoding Kit 24 (SQK-NBD114.24, Oxford Nanopore) and basecalled using Dorado (v7.3.11, e8.2_400bps_sup@v5.0.0 model).

Technical sequences, such as Nanopore adaptors and low-quality reads (≤1,000 bp, Q ≤10), were removed by Porechop ABI (v0.50, ABI mode) (5) and Chopper (v0.80) (6), respectively. Genomes were assembled with Flye (v2.9.3; nano-hq mode) (7) and polished with Medaka (v1.11.3). Genome completeness and contamination were calculated using CheckM2 (v1.0.1) (8). Genomes with ≥99.90% ANI were dereplicated using OrthoANIu (9) and annotated using NCBI Prokaryotic Genome Annotation Pipeline (PGAP, version 6.7) (10), and CRISPR regions were identified using CRISPR-CasFinder (subtype clustering model) (11). Taxonomy was assigned using GTDB-Tk (v2.4.0 with release 220 database) (12).

The nine isolate genomes with fully circularized chromosomes, determined by the Flye assembler, ranged in size from 3,939,831 to 7,333,835 base pairs, encoding 3,610 to 6,553 coding sequences (CDS) and 72 to 118 tRNAs (Table 1). Extrachromosomal elements (ECEs) were present in four genomes, with the RS8 genome containing six ECEs. Among the isolates, *Lysinibacillus*, *Peribacillus*, and *Paenibacillus* are gram-positive, while *Pseudomonas*, *Stenotrophomonas*, and *Acinetobacter* are gram-negative.

**TABLE 1:** Summary of isolates and their genomes

Isolate ID	LS1	LW8	RS5	RS7	RS8	RS10	RS11	RW6	RW9
Culture media	LB	LB	R2A	R2A	R2A	R2A	R2A	R2A	R2A
Classification	*Lysinibacillus fusiformis*	*Pseudomonas* sp.	*Lysinibacillus* sp.	*Peribacillus* sp.	*Paenibacillus* sp.	*Lysinibacillus* sp.	*Lysinibacillus* sp.	*Acinetobacter* sp.	*Stenotrophomonas lactitubi*
Genome size (bp)	4,761,317	5,952,450	5,165,228	5,809,518	7,333,835	5,165,155	4,918,501	3,939,831	4,537,953
Status	Complete	Complete	Complete	Complete	Complete	Complete	Complete	Complete	Complete
Genome coverage	47×	21×	46×	22×	28×	52×	59×	182×	78×
Number of reads	37,364	15,924	23,584	75,412	115,168	27,450	63,631	81,173	117,616
N50 reads (bp)	18,682	20,361	18,511	10,091	4,146	18,060	14,863	19,989	8,298
GC%	37.6	60.0	37.1	39.8	43.6	37.1	37.0	38.8	66.1
No. of sequences	1	2	1	3	7	1	5	1	1
CDS	4,602	5,277	4,900	5,508	6,547	4,995	4,779	3,651	4,049
tRNA	107	70	109	83	88	109	119	73	72
CRISPR repeats	1	2	14	6	8	14	13	0	8
Plastic degradation genes		PHA depolymerase, lipase, esterase		Oxr1 Oxidoreductase				Phthalate dioxygenase reductase	PHA depolymerase, PHB depolymerase
Hydrocarbon degradation genes		LadA						AlkB (2), LadA, AlmA	
GenBank accession	CP167119	CP168138 (chromosome), CP168137 (pLW8a)	CP169418	CP168135 (chromosome), CP168134 (pRS7b), CP168136 (pRS7a)	CP168131 (chromosome), CP168128 (pRS8c), CP168129 (pRS8d), CP168130 (pRS8a), CP168132 (pRS8b), CP168133 (pRS8e)	CP167122	CP168124 (chromosome), CP168123 (pRS11a), CP168125 (pRS11b), CP168126 (pRS11d), CP168127 (pRS11c)	CP167121	CP167120
SRA accession	SRR31131491	SRR31131486	SRR31131489	SRR31131487	SRR31131488	SRR31131490	SRR31131492	SRR31131493	SRR31131485

As these bacteria were isolated from microplastics, genomes were searched for plastic degradation genes using BLAST against PlasticDB and the Plastic Microbial Biodegradation Database (13, 14). Four genomes had hits for plastic-degrading genes (>80% identity). Isolate LW8 and RW9 have a gene for polyhydroxyalkanoate (PHA) depolymerase that can degrade poly(3-hydroxybutyrate-co-3-hydroxyvalerate), polycaprolactone, polyethersulfone, polyhydroxyalkanoate, and polylactic acid. RW9 also has a polyhydroxybutyrate (PHB) depolymerase gene involved in the biodegradation of polyhydroxybutyrate. RS7 and RW6 contained genes for polyurethane and phthalate biodegradation, respectively. Some plastics are hydrocarbon derivatives and can be degraded through hydrocarbon degradation pathways (15, 16). Hence, the genomes were searched for hydrocarbon metabolism markers using CANT-HYD (17). LW8 and RW6 contained long-chain alkane monooxygenase (LadA), with the latter containing two other alkane metabolism genes (AlkB and AlmA).

## Data Availability

Genomes have been deposited in NCBI GenBank under Bioproject PRJNA1145385.

## References

[1] Zettler ER, Mincer TJ, Amaral-Zettler LA. 2013. Life in the “plastisphere”: microbial communities on plastic marine debris. Environ Sci Technol 47:7137–7146. doi:10.1021/es401288x 23745679

[2] Stadfeld J, Suresh S, Bhatnagar S. 2024. Non-destructive microplastic isolation from water and sediment samples v1. Protocols.io. doi:10.17504/protocols.io.4r3l2q264l1y/v1

[3] Bertani G. 1951. Studies on lysogenesis I. J Bacteriol 62:293–300. doi:10.1128/jb.62.3.293-300.1951 14888646 PMC386127

[4] Reasoner DJ, Geldreich EE. 1985. A new medium for the enumeration and subculture of bacteria from potable water. Appl Environ Microbiol 49:1–7. doi:10.1128/aem.49.1.1-7.1985 3883894 PMC238333

[5] Bonenfant Q, Noé L, Touzet H. 2023. Porechop_ABI: discovering unknown adapters in Oxford Nanopore Technology sequencing reads for downstream trimming. Bioinform Adv 3:vbac085. doi:10.1093/bioadv/vbac085 36698762 PMC9869717

[6] De Coster W, Rademakers R. 2023. NanoPack2: population-scale evaluation of long-read sequencing data. Bioinformatics 39:btad311. doi:10.1093/bioinformatics/btad311 37171891 PMC10196664

[7] Kolmogorov M, Yuan J, Lin Y, Pevzner PA. 2019. Assembly of long, error-prone reads using repeat graphs. Nat Biotechnol 37:540–546. doi:10.1038/s41587-019-0072-8 30936562

[8] Chklovski A, Parks DH, Woodcroft BJ, Tyson GW. 2024. Author correction: checkM2: a rapid, scalable and accurate tool for assessing microbial genome quality using machine learning. Nat Methods 20:1203–1212. doi:10.1038/s41592-023-01940-w 37500759

[9] Yoon S-H, Ha S-M, Lim J, Kwon S, Chun J. 2017. A large-scale evaluation of algorithms to calculate average nucleotide identity. Antonie Van Leeuwenhoek 110:1281–1286. doi:10.1007/s10482-017-0844-4 28204908

[10] Tatusova T, DiCuccio M, Badretdin A, Chetvernin V, Nawrocki EP, Zaslavsky L, Lomsadze A, Pruitt KD, Borodovsky M, Ostell J. 2016. NCBI prokaryotic genome annotation pipeline. Nucleic Acids Res 44:6614–6624. doi:10.1093/nar/gkw569 27342282 PMC5001611

[11] Couvin D, Bernheim A, Toffano-Nioche C, Touchon M, Michalik J, Néron B, Rocha EPC, Vergnaud G, Gautheret D, Pourcel C. 2018. CRISPRCasFinder, an update of CRISRFinder, includes a portable version, enhanced performance and integrates search for Cas proteins. Nucleic Acids Res 46:W246–W251. doi:10.1093/nar/gky425 29790974 PMC6030898

[12] Chaumeil P-A, Mussig AJ, Hugenholtz P, Parks DH. 2022. GTDB-Tk v2: memory friendly classification with the genome taxonomy database. Bioinformatics 38:5315–5316. doi:10.1093/bioinformatics/btac672 36218463 PMC9710552

[13] Gambarini V, Pantos O, Kingsbury JM, Weaver L, Handley KM, Lear G. 2022. PlasticDB: a database of microorganisms and proteins linked to plastic biodegradation. Database 2022:baac008. doi:10.1093/database/baac008 35266524 PMC9216477

[14] Gan Z, Zhang H. 2019. PMBD: a comprehensive plastics microbial biodegradation database. Database 2019:baz119. doi:10.1093/database/baz119 31738435 PMC6859810

[15] Mandal M, Roy A, Popek R, Sarkar A. 2024. Micro- and nano- plastic degradation by bacterial enzymes: a solution to “White Pollution”. The Microbe 3:100072. doi:10.1016/j.microb.2024.100072

[16] Mohanan N, Montazer Z, Sharma PK, Levin DB. 2020. Microbial and enzymatic degradation of synthetic plastics. Front Microbiol 11:580709. doi:10.3389/fmicb.2020.580709 33324366 PMC7726165

[17] Khot V, Zorz J, Gittins DA, Chakraborty A, Bell E, Bautista MA, Paquette AJ, Hawley AK, Novotnik B, Hubert CRJ, Strous M, Bhatnagar S. 2021. CANT-HYD: a curated database of phylogeny-derived hidden markov models for annotation of marker genes involved in hydrocarbon degradation. Front Microbiol 12:764058. doi:10.3389/fmicb.2021.764058 35069469 PMC8767102

